# Supplementary material for: Orthogonal control of mean and variability of endogenous genes in a human cell line
Source: Nat Commun. 2021 Jan 12;12:292. doi: 10.1038/s41467-020-20467-8 (PMC7804932; doi:10.1038/s41467-020-20467-8)
Supplement: Supplementary file 1 — Description of Additional Supplementary Files [file 41467_2020_20467_MOESM1_ESM.pdf]

Title: Supplementary Data 1

Description: Full list of padj values provided as .xls file
