# Supplementary material for: Orthogonal control of mean and variability of endogenous genes in a human cell line
Source: Nat Commun. 2021 Jan 12;12:292. doi: 10.1038/s41467-020-20467-8 (PMC7804932; doi:10.1038/s41467-020-20467-8)
Supplement: Supplementary file 2 — Supplementary Information [file 41467_2020_20467_MOESM2_ESM.pdf]

Supplementary Information to:

Orthogonal Control of Mean and Variability of Endogenous Genes in a Human Cell Line

Bonny *et al*.

SUPPLEMENTARY INFORMATION

Supplemental Data

Supplementary Data 1 Full list of padj values provided as .xls file

Supplementary Tables

Supplementary Table 1 List of plasmids used and corresponding figures

Supplementary Table 2 List of primers, Cas9 protospacer sequences

Supplementary Figures

Supplementary Figure 1 TuNR circuit characterization

Supplementary Figure 2 Orthogonality and multiplier effect of TuNR nodes

Supplementary Figure 3 Scatter plots of TuNR expression for tagBFP and mAzamiGreen

Supplementary Figure 4 Broader examination of attainable TuNR isomeans

Supplementary Figure 5 Reproducibility of mean and CV^2 from TuNR

Supplementary Figure 6 TuNR chassis cell line characterization

Supplementary Figure 7 Reproducibility of mean and CV^2 from TuNR at endogenous genes

Supplementary Figure 8 Cumulative Density Functions of TuNR between independent replicates

Supplementary Data 1: **All associated adjusted p values for distributions examined**

Page 1 contains associated well name, inducer concentrations and p values for independent replicates of clone A10, both mAzamiGreen and tagBFP. Page 2 contains associated well name, inducer concentrations and p values for independent replicates of clone E9, both mAzamiGreen and tagBFP. Page 3 contains associated endogenous target, well name, inducer concentrations and p values for independent replicates of clone F11. Page 4 contains associated p values for representative distributions found in Figure 2D. Page 5 contains associated p values for representative distributions found in Figure 3G-H.

Supplementary Figure 1: **TuNR nodes are orthogonal and circuit has low basal expression of terminal output.** a) Full circuit diagram of TuNR targeted towards a pTRE promoter driving mAzamiGreen expression. b) Cells for all analyses were gated based on FSC and SSC and then for iRFP expression. c) Expression of mRuby from TuNR clones A10 (left) and E9 (right) induced with increasing concentrations of ABA over 7 days. Media was replenished every 24 hours. d) Distributions of mRuby expression on day 3 with increasing doses of ABA (shades of red). Data are taken at day 3 when the means have reached steady-state (Figure 1B in the main text). e) Time-dependent mAzamiGreen expression from TuNR clones A10 (left) and E9 (right). Cells were first induced with 400 μM of ABA for 3 days, at which increasing concentrations of GA were added. Measurements were carried out over the following 7 days while keeping ABA and GA concentration constant through daily replenishment. f) Distributions of the expression of mAzamiGreen representing the abundance of dCas9 with (red) and without (beige) full induction of ABA. g) mRuby expression plotted as a function of ABA concentration with increasing amounts of GA (concentration increasing with shade of green). These are the same data as shown in Figure 1D in the main text.

Supplementary Figure 2: **First node of TuNR is responsive only to ABA, and second node is responsive to both ABA and GA.** a) Heatmap of steady-state mRuby expression in response to the activation of the first node of TuNR with increasing ABA concentration (y-axis), and increasing GA (x-axis). b) Heatmaps of steady-state mAzamiGreen (left) and c) tagBFP (right) expression with increasing ABA (y-axis), and increasing GA (x-axis).

Supplementary Figure 3: **Correlated expression of terminal outputs mAzamiGreen and tagBFP across 96 combinations of ABA and GA.** a) Representative scatter plots of tagBFP and mAzamiGreen expression from TuNR induced with 96 combinations of ABA and GA.

Supplementary Figure 4: **TuNR achieves several isomean combinations of inducers with different variability.** a) Heatmap of mean and b) CV^2 of mAzamiGreen expression. c) Example mAzamiGreen distributions binned by the same mean expression. d) Heatmap of mean and e) CV^2 of tagBFP expression. f) Example tagBFP distributions binned by the same mean expression.

Supplementary Figure 5: **Mean expression and CV^2 from terminal nodes mAzamiGreen and tagBFP are strongly correlated across independent replicates and independent clones.** a) All-by-all plot of mean expression by well between mAzamiGreen and tagBFP, and across independent replicates and different clones of TuNR. R, Pearson Correlation Coefficient. Each point represents a different concentration of ABA/GA induction. b) All-by-all plot of well CV^2 between mAzamiGreen and tagBFP, and across independent replicates and different clones of TuNR. Each point represents a different concentration of ABA/GA induction.

Supplementary Figure 6: **TuNR "chassis" clonal cell lines reach steady state with comparable on-kinetics to previous TuNR cell lines.** a) Time-dependent mRuby expression

of three independent clones, E11 (left), F5 (center), F11 (right) induced with increasing concentrations of ABA (shades of red). Error bars represent the 95% Confidence Interval of the mean of N = 1000 cells from each clone. b) Clone F11 distributions of mRuby expression at steady-state (after 3 days) per dosage of ABA (shades of red). c) Heatmap of the mean expression of *NGFR* and d) *CXCR4* in the clone F11 background.

Supplementary Figure 7: **Trends of TuNR-mediated mean and variability control are maintained across independent replicates.** a) Plot of the 96 combinations of ABA and GA population mean expression of *NGFR* and b) *CXCR4* between independent replicates. R, Pearson Correlation Coefficient. c) Plot of the corresponding CV^2 for the same 96 combinations of ABA and GA for *NGFR* and d) *CXCR4* independent replicates.

Supplementary Figure 8: **Cumulative density functions of TuNR-mediated distributions show robust reproducibility across independent replicates.** a) Cumulative density functions of all inducer concentrations in Clone A10, mAzamiGreen, b) Clone A10, tagBFP, c) Clone E9, mAzamiGreen, d) Clone E9, tagBFP where replicate 1(blue trace) and replicate 2(orange trace) are overlaid. Grey boxes represent distributions of $P_{adj} < 0.05$; two-sided Kolmorgorov-Smirnov test (Bonferroni corrected). e) Cumulative density functions of endogenous genes *CXCR4* and f) *NGFR*, where replicate 1 (blue trace) and replicate 2 (red trace) are overlaid. Grey boxes represent distributions of $P_{adj} < 0.05$; two-sided Kolmorgorov-Smirnov test (Bonferroni corrected). Full list of inducer concentrations, Kolmorgorov-Smirnov statistics and associated Padj values found in Supplementary Data 1 .xls file.

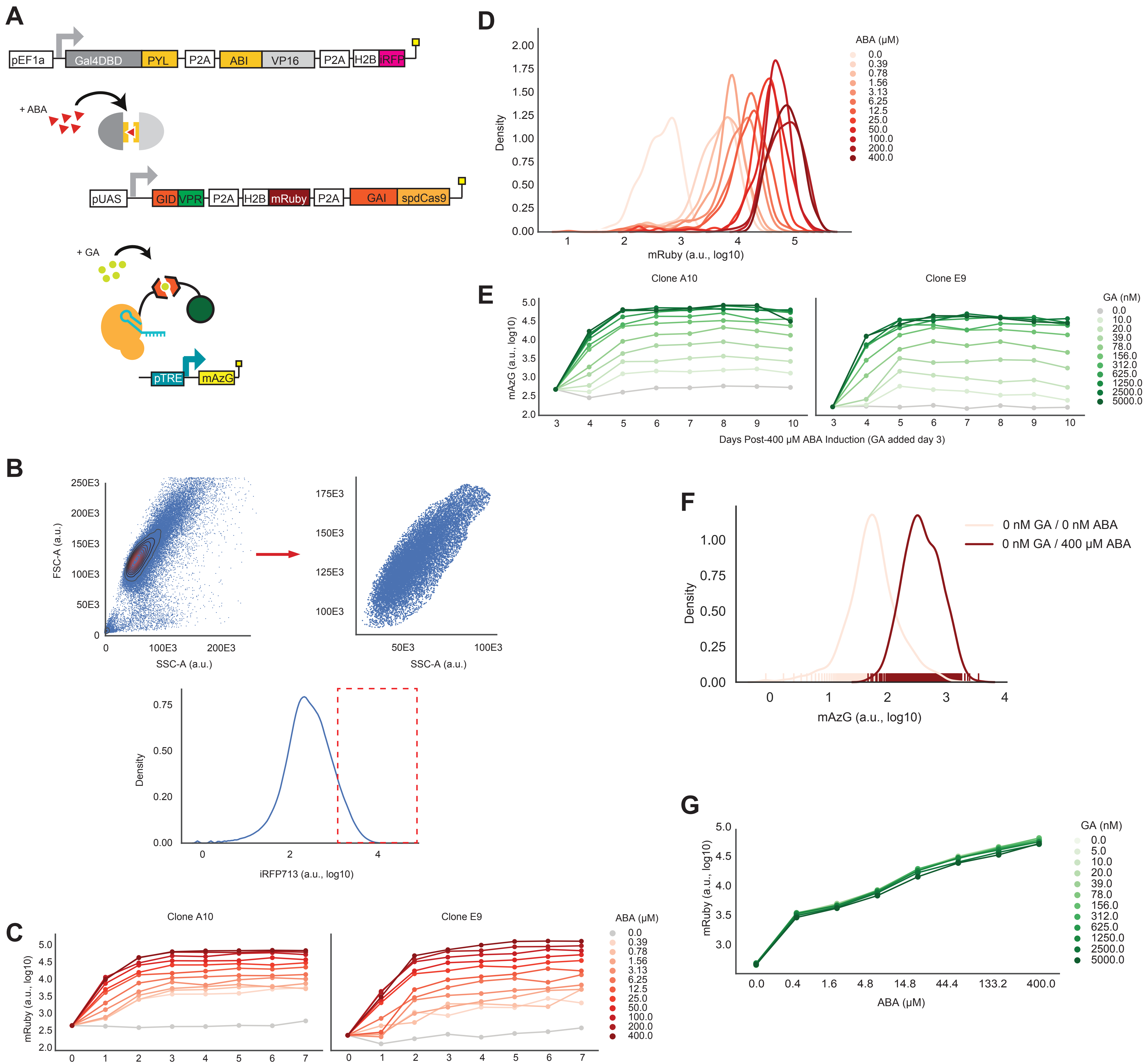

Supplementary Figure 1

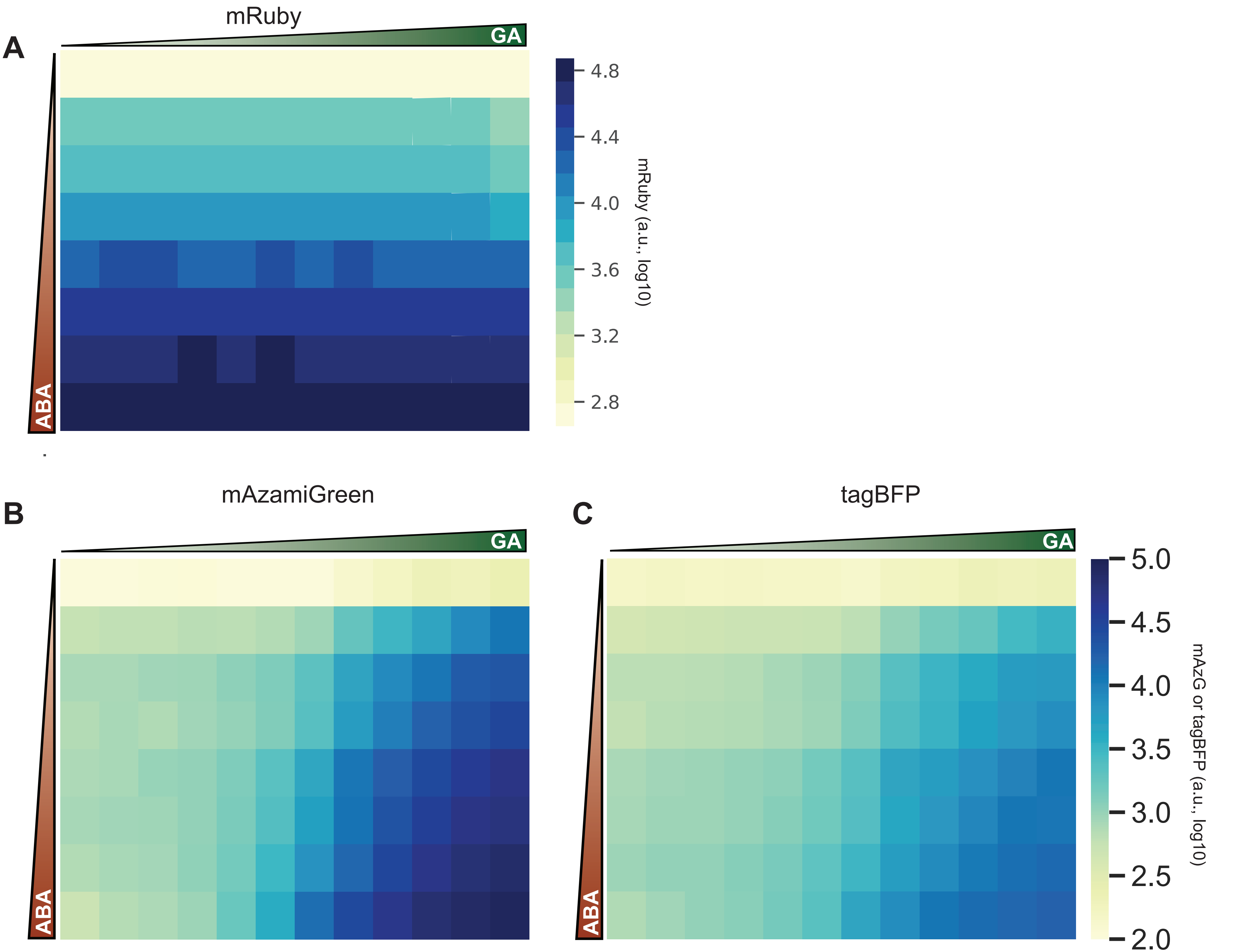

Supplementary Figure 2

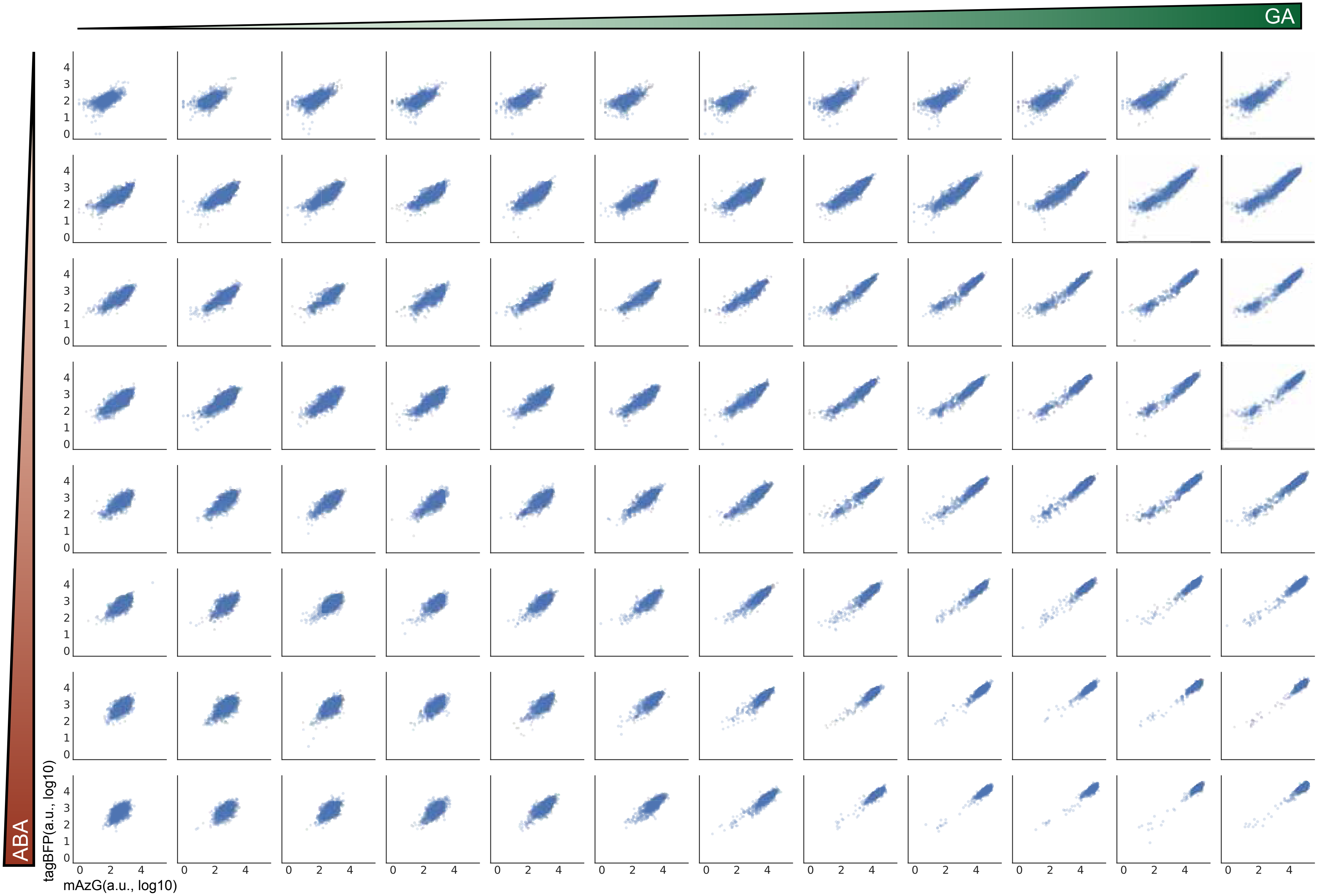

Supplementary Figure 3

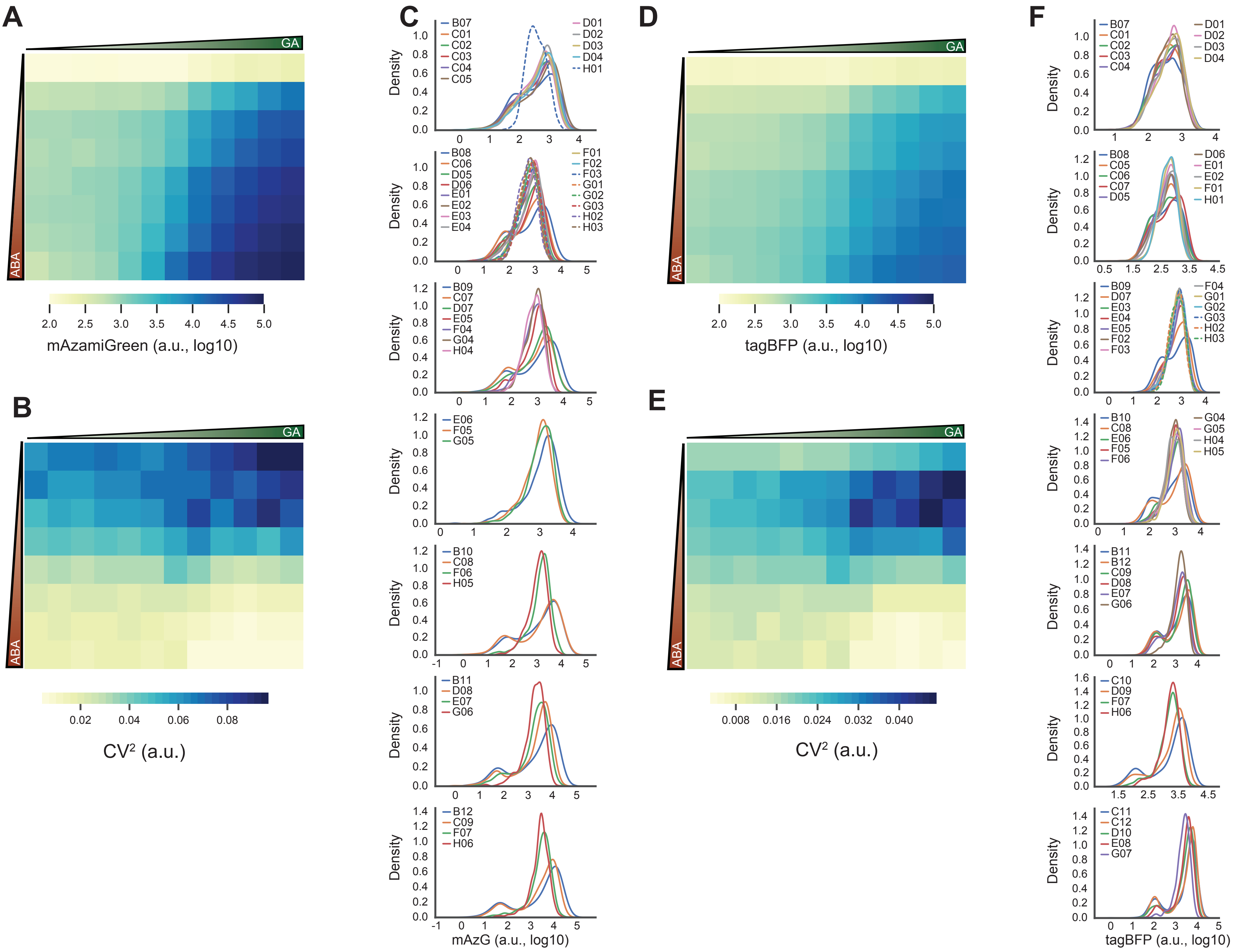

Supplementary Figure 4

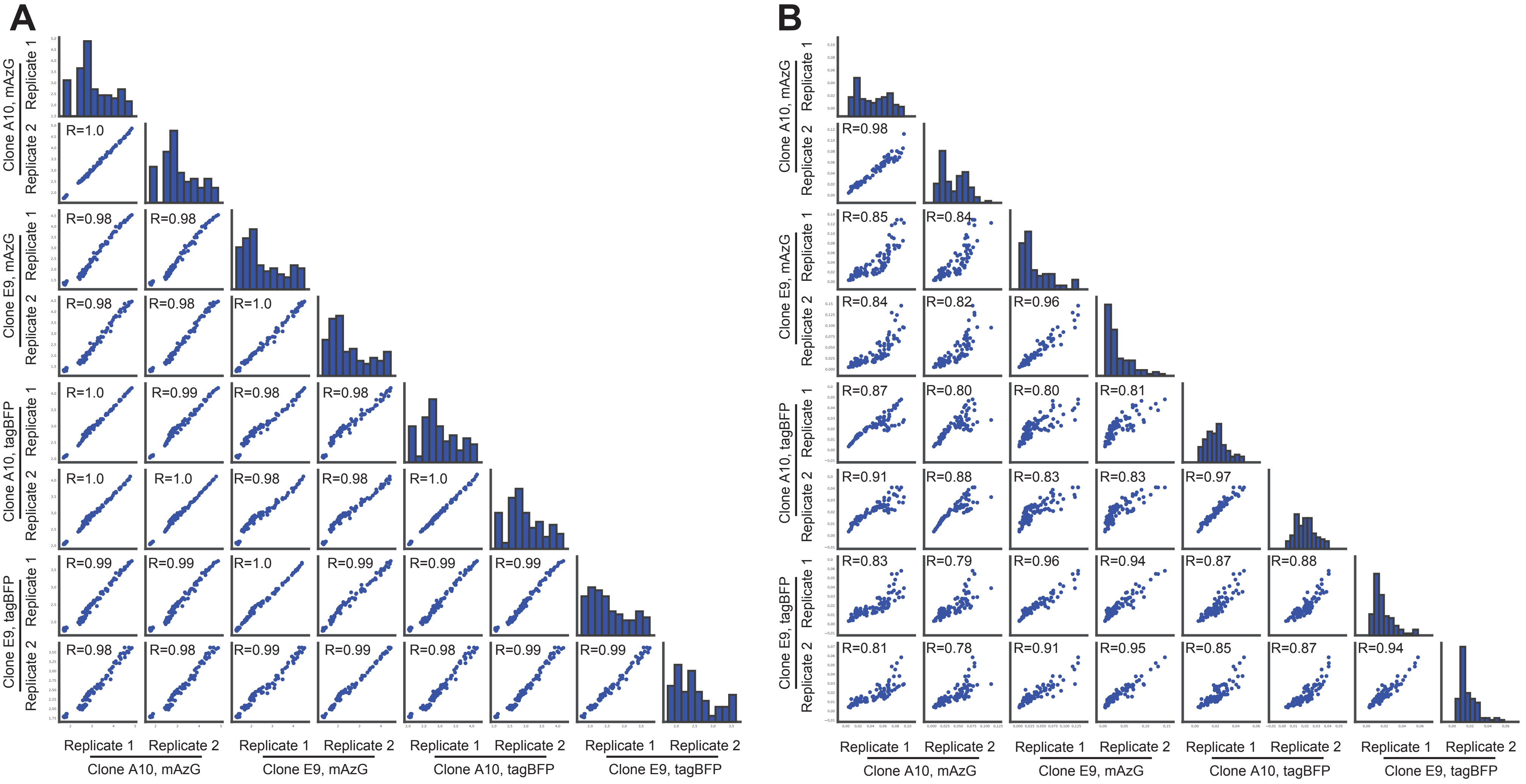

Supplementary Figure 5

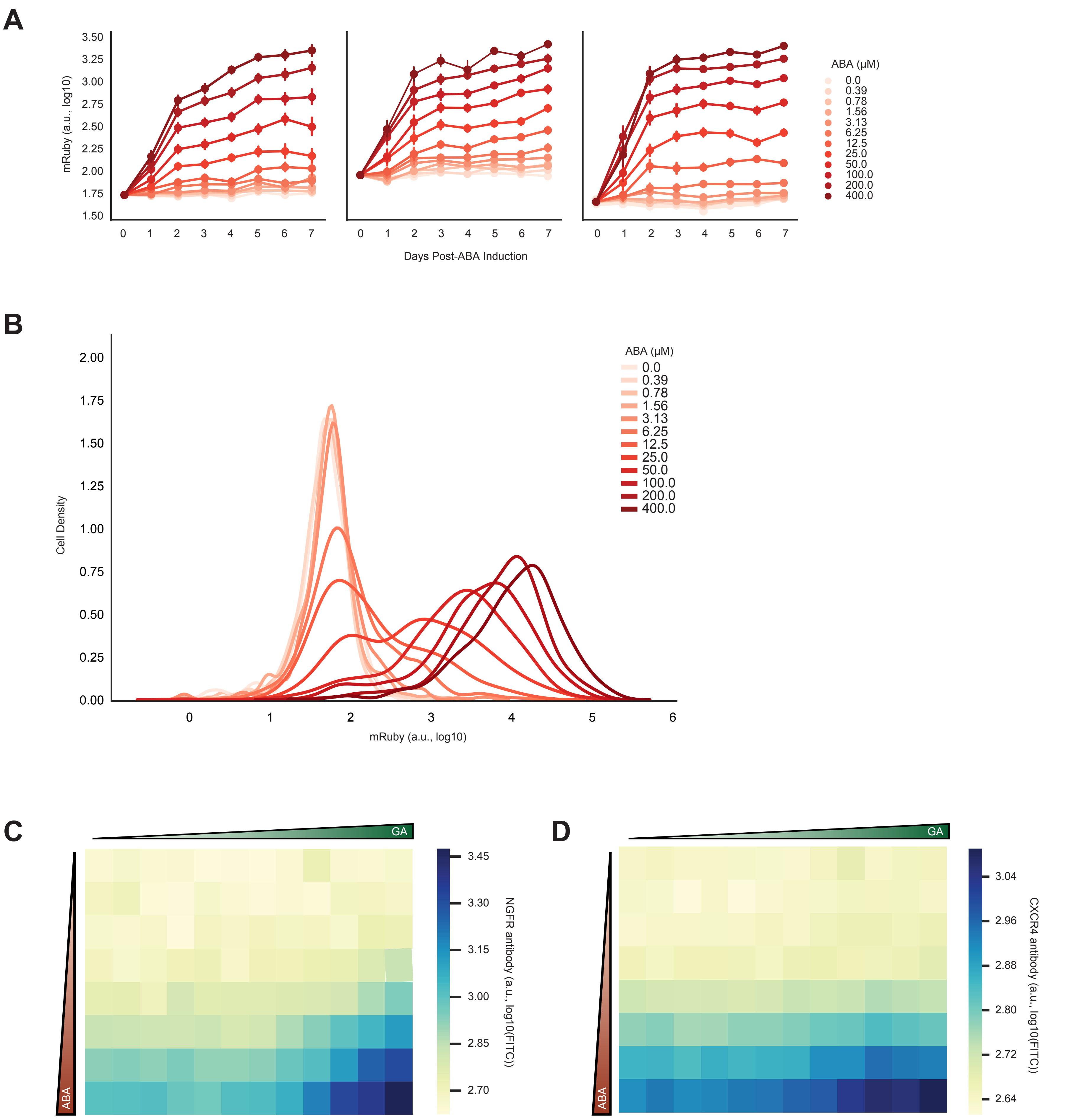

Supplementary Figure 6

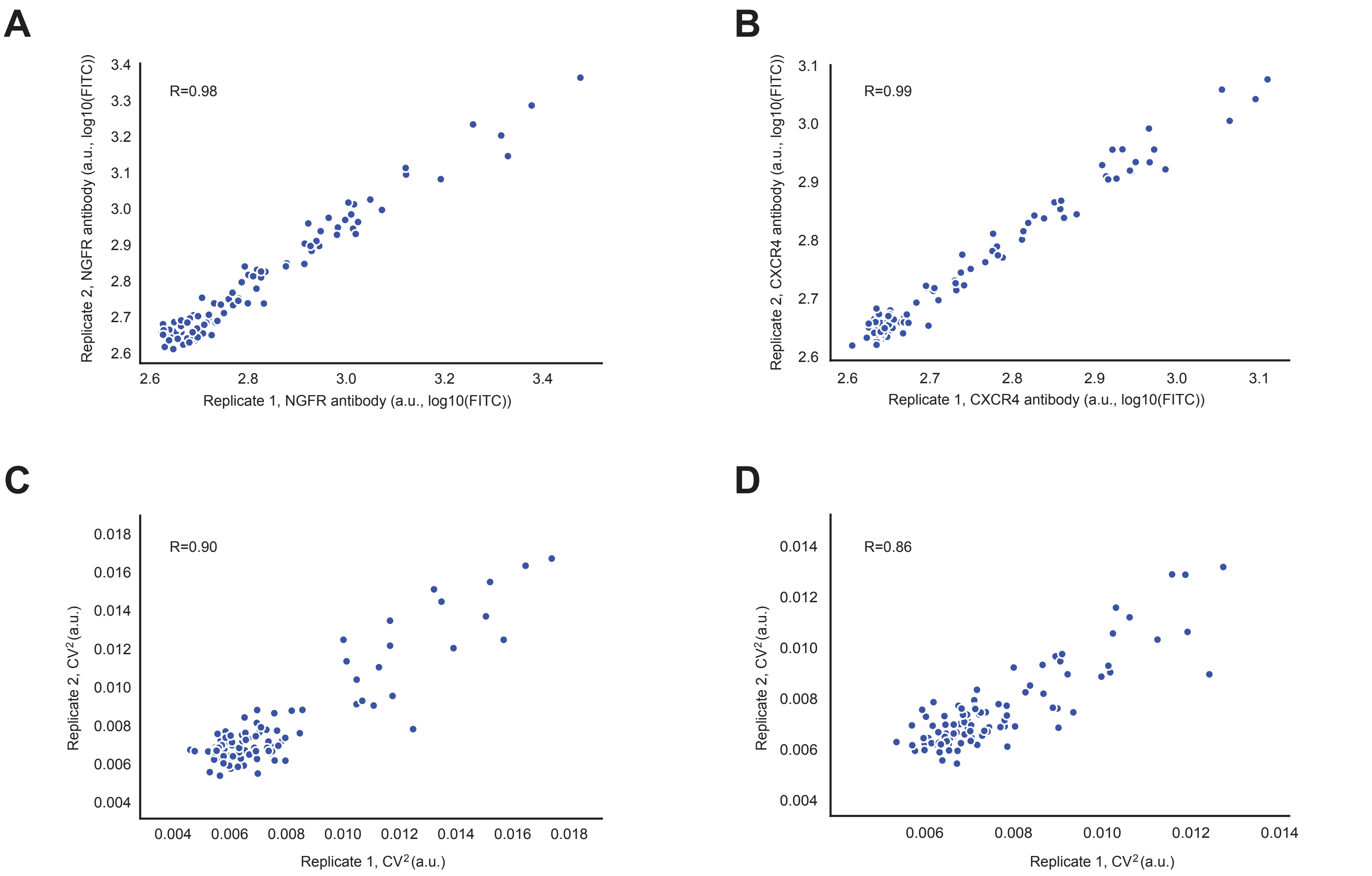

Supplementary Figure 7

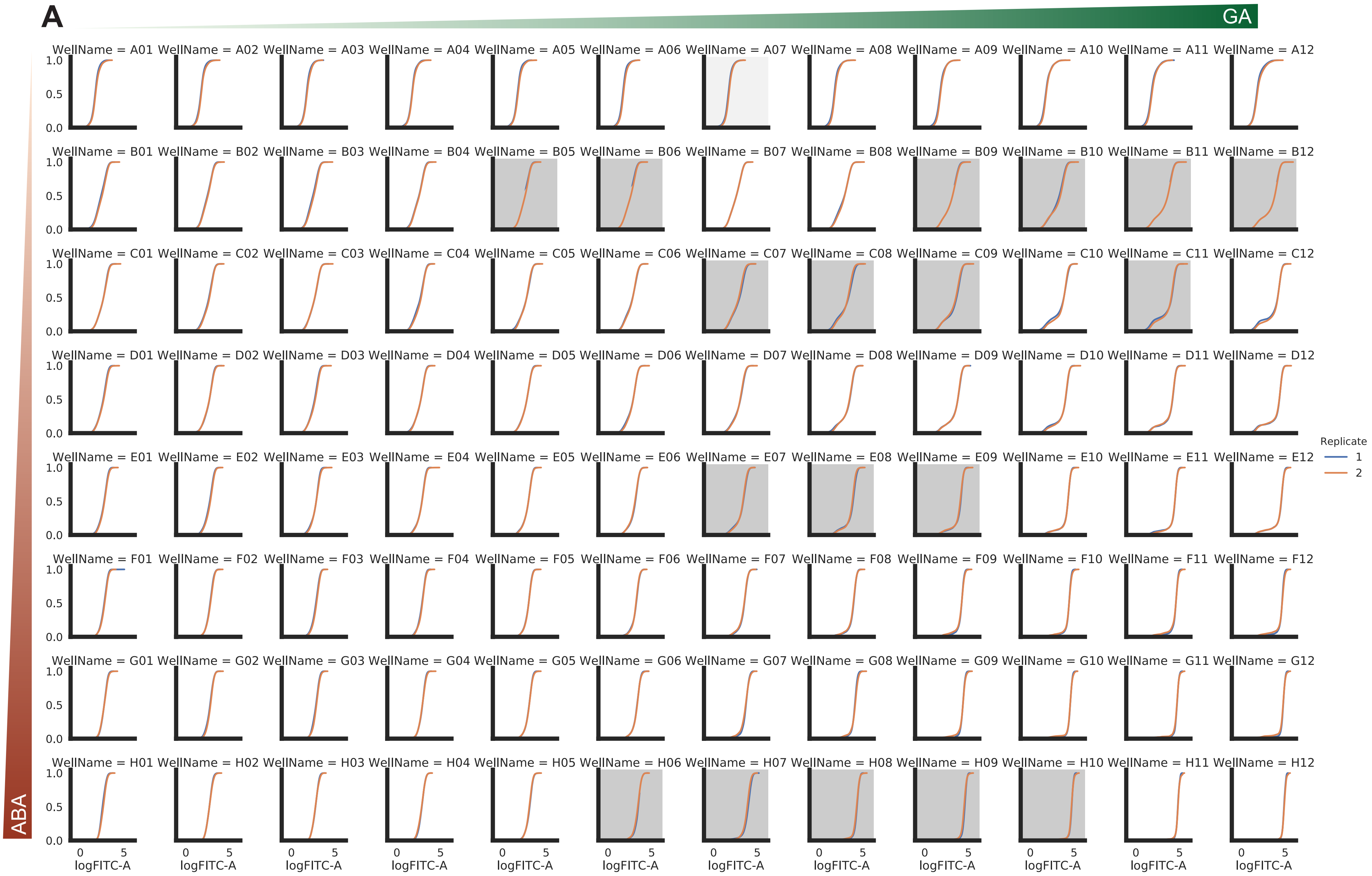

Supplementary Figure 8

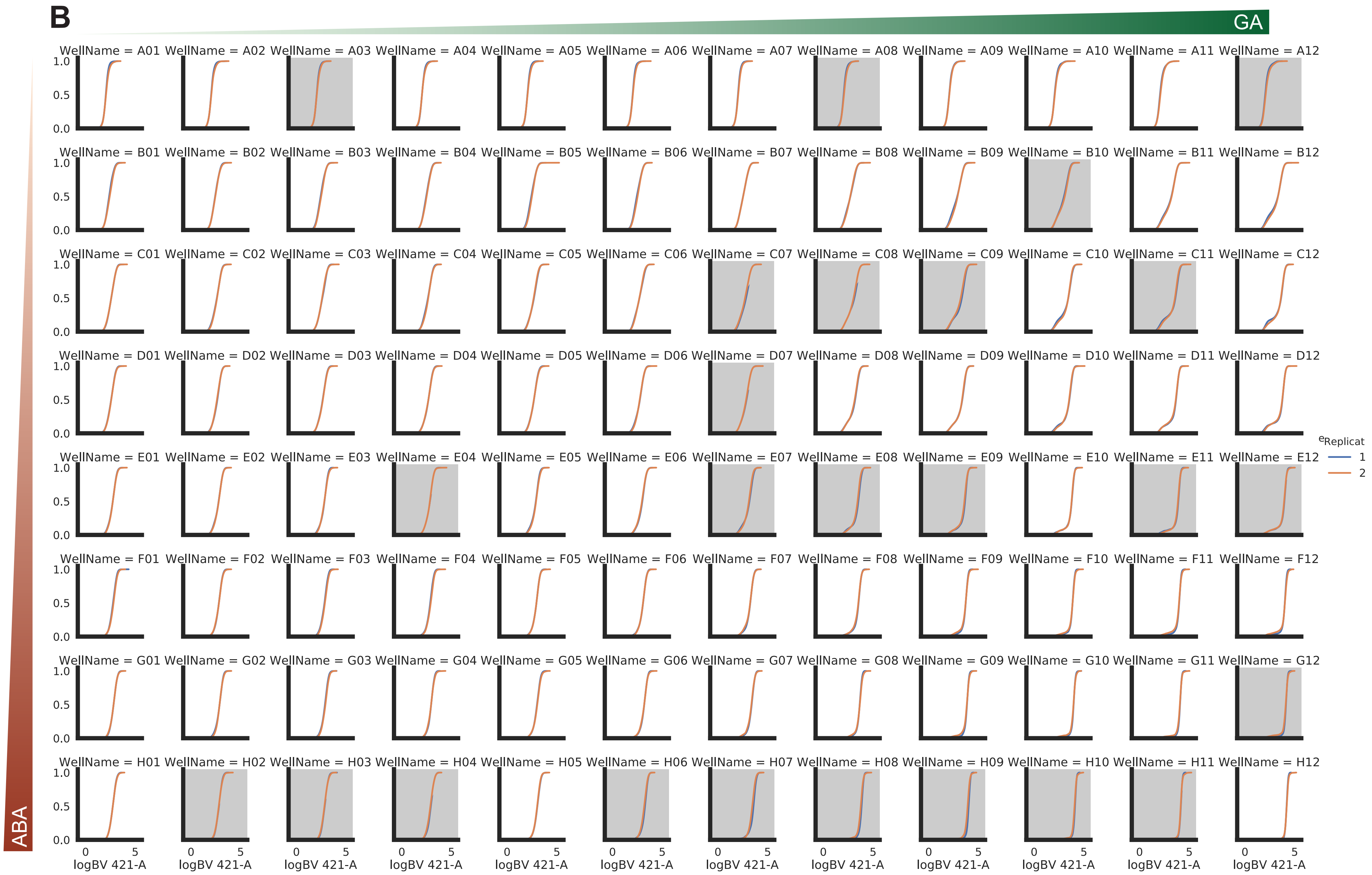

Supplementary Figure 8

C

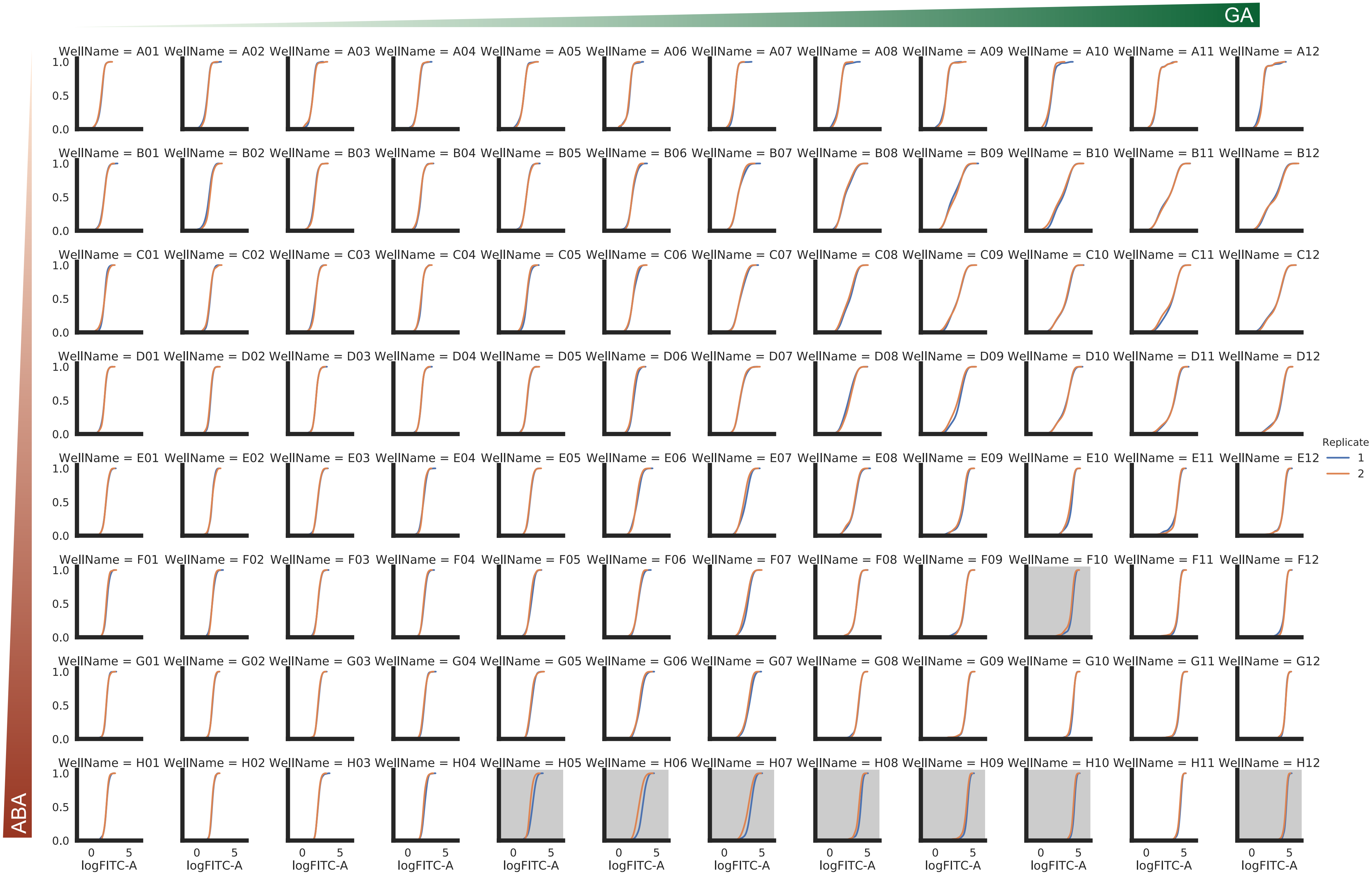

Supplementary Figure 8

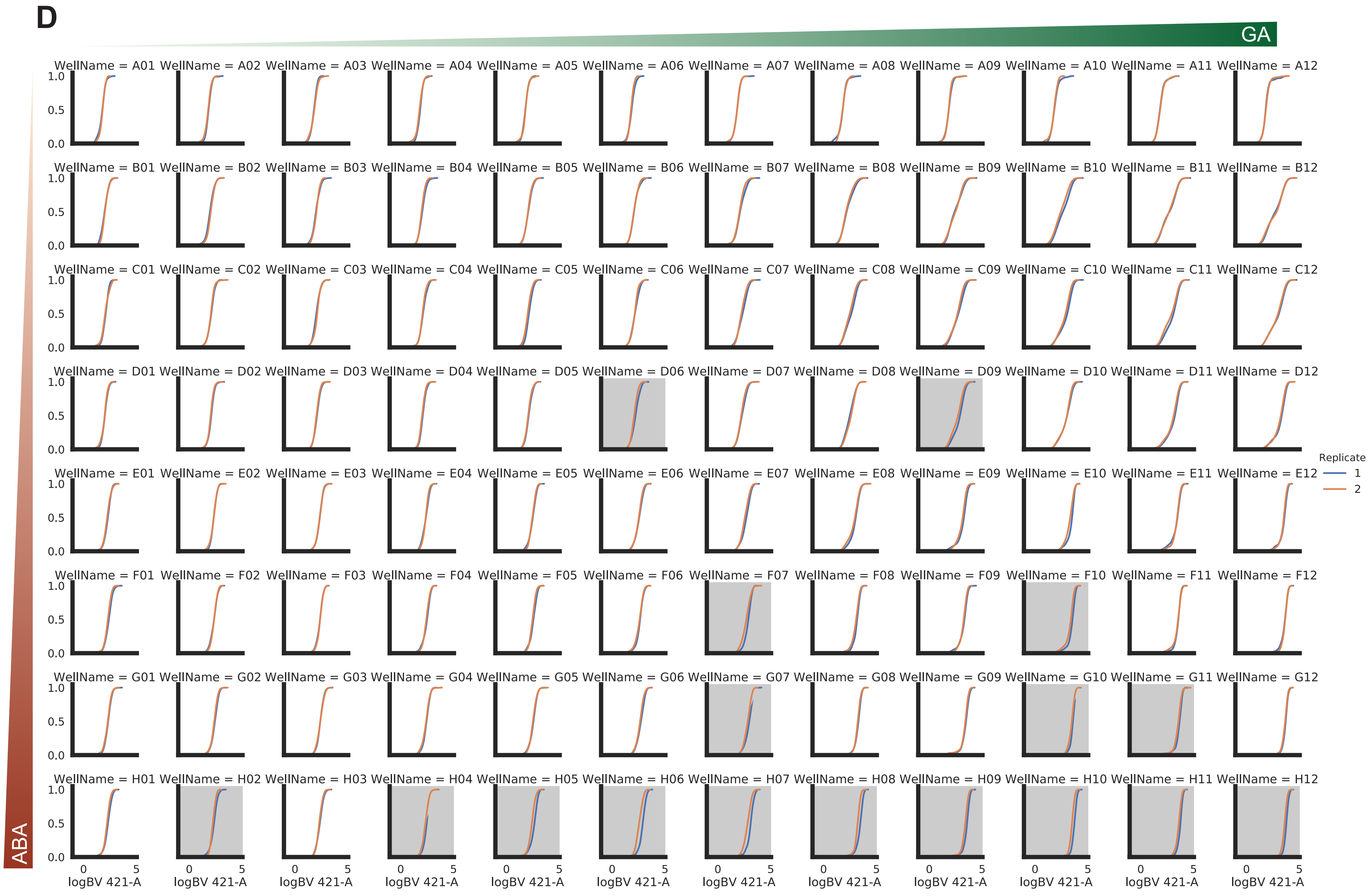

Supplementary Figure 8

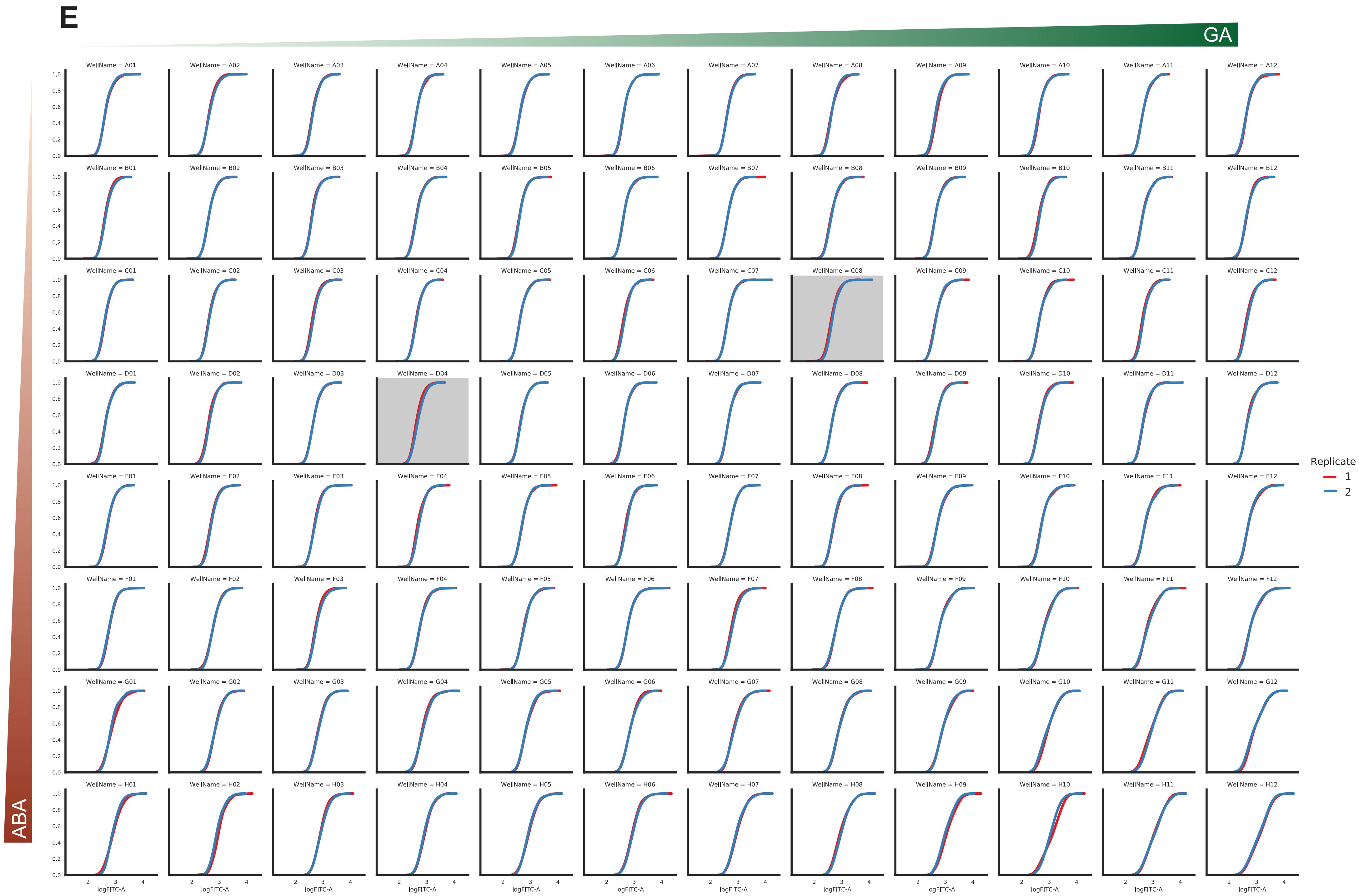

Supplementary Figure 8

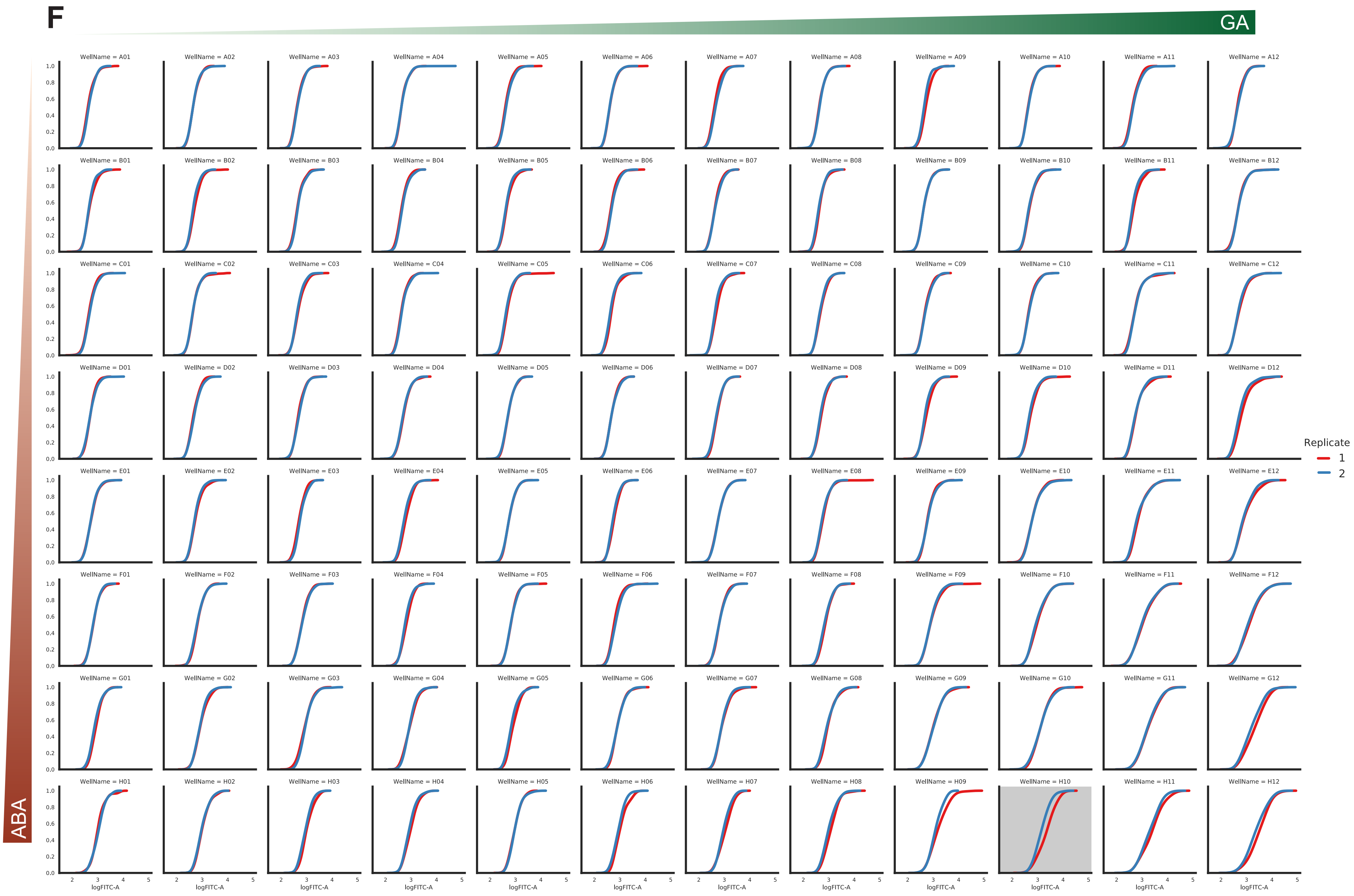

Supplementary Figure 8
