# Supplementary material for: Orthogonal control of mean and variability of endogenous genes in a human cell line
Source: Nat Commun. 2021 Jan 12;12:292. doi: 10.1038/s41467-020-20467-8 (PMC7804932; doi:10.1038/s41467-020-20467-8)
Supplement: Supplementary file 4 — Reporting Summary [file 41467_2020_20467_MOESM4_ESM.pdf]

nature research

Corresponding author(s): Hana El-Samad
Last updated by author(s): Nov 17, 2020

Reporting Summary

Nature Research wishes to improve the reproducibility of the work that we publish. This form provides structure for consistency and transparency in reporting. For further information on Nature Research policies, see our Editorial Policies and the Editorial Policy Checklist.

Statistics

For all statistical analyses, confirm that the following items are present in the figure legend, table legend, main text, or Methods section.

n/a	Confirmed	
[ ]	[x]	The exact sample size (n) for each experimental group/condition, given as a discrete number and unit of measurement
[ ]	[x]	A statement on whether measurements were taken from distinct samples or whether the same sample was measured repeatedly
[ ]	[x]	The statistical test(s) used AND whether they are one- or two-sided<br>*Only common tests should be described solely by name; describe more complex techniques in the Methods section.*
[x]	[ ]	A description of all covariates tested
[ ]	[x]	A description of any assumptions or corrections, such as tests of normality and adjustment for multiple comparisons
[ ]	[x]	A full description of the statistical parameters including central tendency (e.g. means) or other basic estimates (e.g. regression coefficient) AND variation (e.g. standard deviation) or associated estimates of uncertainty (e.g. confidence intervals)
[ ]	[x]	For null hypothesis testing, the test statistic (e.g. F, t, r) with confidence intervals, effect sizes, degrees of freedom and P value noted<br>*Give P values as exact values whenever suitable.*
[x]	[ ]	For Bayesian analysis, information on the choice of priors and Markov chain Monte Carlo settings
[x]	[ ]	For hierarchical and complex designs, identification of the appropriate level for tests and full reporting of outcomes
[ ]	[x]	Estimates of effect sizes (e.g. Cohen's d, Pearson's r), indicating how they were calculated

Our web collection on statistics for biologists contains articles on many of the points above.

Software and code

Policy information about availability of computer code

Data collection	Data was acquired on an LSR Fortessa and a Nikon Ti Inverted Widefield Epifluorescence microscope.
Data analysis	All analysis of flow cytometry data was performed using the FlowCytometryTools v0.5.0 and SciPy v1.1.0 package in Python 2.7. Images were processed using Fiji (ImageJ)

For manuscripts utilizing custom algorithms or software that are central to the research but not yet described in published literature, software must be made available to editors and reviewers. We strongly encourage code deposition in a community repository (e.g. GitHub). See the Nature Research guidelines for submitting code & software for further information.

Data

Policy information about availability of data

All manuscripts must include a data availability statement. This statement should provide the following information, where applicable:
- Accession codes, unique identifiers, or web links for publicly available datasets
- A list of figures that have associated raw data
- A description of any restrictions on data availability

All source data is available at https://ucsf.box.com/s/he29gcnt6igblwo56jvtgg4p34k2gzrg.

Field-specific reporting

Life sciences study design

All studies must disclose on these points even when the disclosure is negative.

Sample size	No sample size calculation was performed. Independent replicates are of the same cell line that were independently induced, treated and brought to cytometry. Additionally, two independent clones of cell lines shown in figure 2 for a total of four replicates. The rationale for sample sizes was that between clones with different genomic integrations and replicates of those clones, the trends were sufficiently reproducible.
Data exclusions	No data was excluded.
Replication	All experimental findings were reproduced successfully using independent replicates, as described in figure captions. Data displaying correlations between replicates is provided in the supplemental figures
Randomization	Randomization was not performed in this study because experiments were generated from the same parental cell line prior to transfection.
Blinding	Blinding was not performed in this study because experiments were carried out by individual researchers.

Reporting for specific materials, systems and methods

We require information from authors about some types of materials, experimental systems and methods used in many studies. Here, indicate whether each material, system or method listed is relevant to your study. If you are not sure if a list item applies to your research, read the appropriate section before selecting a response.

Materials & experimental systems

n/a	Involved in the study	
☐	☒	Antibodies
☐	☒	Eukaryotic cell lines
☒	☐	Palaeontology and archaeology
☒	☐	Animals and other organisms
☒	☐	Human research participants
☒	☐	Clinical data
☒	☐	Dual use research of concern

Methods

n/a	Involved in the study	
☒	☐	ChIP-seq
☐	☒	Flow cytometry
☒	☐	MRI-based neuroimaging

Antibodies

Antibodies used	CXCR4: #53-9991-80 from ThermoFisher; NGFR: #345104 from BioLegend
Validation	From BioLegend: "Each lot of this antibody is quality control tested by immunofluorescent staining with flow cytometric analysis." This antibody was raised in mouse and tested against Human neuroblastoma cell line SK-N-MC. From ThermoFisher: "This 2B11 antibody has been tested by flow cytometric analysis of mouse thymocytes." This antibody was raised in Rat and tested against C57Bl/6 thymocytes CD184 (CXCR4). Antibody is use against both mouse and human CXCR4.

Eukaryotic cell lines

Policy information about cell lines

Cell line source(s)	PC-9 cell line was a gift from the labs of Lani Wu and Steven Altschuler. HEK LX293T was a gift from the lab of Wendell Lim. Both cell lines were originally purchased from ATCC
Authentication	HEK LX293T cells were assessed for their hallmark morphology under the microscope. Both cell lines were otherwise not authenticated.
Mycoplasma contamination	PC-9 cells gave no indication of Mycoplasma contamination, and HEK LX293T tested negative.
Commonly misidentified lines (See ICLAC register)	Commonly misidentified cell lines were not used in this study.

Flow Cytometry

Plots

Confirm that:

- [x] The axis labels state the marker and fluorochrome used (e.g. CD4-FITC).
- [x] The axis scales are clearly visible. Include numbers along axes only for bottom left plot of group (a 'group' is an analysis of identical markers).
- [x] All plots are contour plots with outliers or pseudocolor plots.
- [x] A numerical value for number of cells or percentage (with statistics) is provided.

Methodology

Sample preparation	Cell cultures featured in Figures 1 and 2 were treated with trypsin and diluted in PBS for flow cytometry experiments. Cultures in Figure 3 was treated with Versene and diluted in 10% FBS in PBS before cytometry.
Instrument	LSR Fortessa (BD)
Software	All analysis of flow cytometry data was performed using the FlowCytometryTools v0.5.0 and SciPy v1.1.0 package in Python 2.7. Microscopy images were processed using Fiji (ImageJ)
Cell population abundance	Approximately 10,000 cells were initially gated for analysis, of which > 90% was used for downstream analysis based on viability as determined by FSC and SSC contour density illustrated in Supplementary Figure 1B.
Gating strategy	We developed a custom python script to establish a contour gate bounded by FSC (50E3-150E3) and SSC (20E3-100E3). Then, using a scatter plot of SSC-A versus SSC-H, we gated on cells that fell along the diagonal (removal of doublets). For all subsequent analyses, a gate of 3000 iRFP713 (a.u.) was applied (presence of circuit). No other gates were applied.

- [x] Tick this box to confirm that a figure exemplifying the gating strategy is provided in the Supplementary Information.
